# Nurturing ethical insight: exploring nursing students’ journey to ethical competence

**DOI:** 10.1186/s12912-024-02243-x

**Published:** 2024-08-15

**Authors:** Sylvia Hansen, Elisabeth Hessevaagbakke, Katrin Lindeflaten, Kaja Elvan, Daniela Lillekroken

**Affiliations:** Department of Nursing and Health Promotion, Oslo Metropolitan University, PB 4 St. Olavs plass N, Oslo, 0130 Norway

**Keywords:** Care values, Clinical placement, Ethical competence, Nursing students, Nursing homes

## Abstract

**Background:**

Ethical competence is a key competence in nursing and the development of the competence is a central part in nursing education. During clinical studies, nursing students face ethical problems that require them to apply and develop their ethical knowledge and skills. Little is known about how ethical competence evolves during students’ initial clinical placements. This study explored the development of ethical competence in first-year nursing students during their first clinical placements in nursing homes.

**Methods:**

This exploratory-descriptive qualitative study used focus group interviews to collect data and a phenomenological hermeneutical method for analysis. Twenty-eight first-year nursing students participated in six focus groups. The data were collected between March and April 2024 at Oslo Metropolitan University in Norway.

**Results:**

The naïve reading of the data involved an awareness of the students applying their prior knowledge, modifying their prior knowledge and developing skills that allowed them to manoeuvre ethical practices that, in some cases, appeared excellent and, in other cases, grim. The structural analysis identified three themes: (i) ethical competence forges in practice, (ii) ethical competence evolves at the intersection of knowledge and skills and (iii) ethical competence unfolds through meaningful discussions. A comprehensive understanding of the data was formulated as ‘Being on a journey towards ethical competence’. This presents a metaphor illustrating that nursing students embark on a journey towards ethical competence; from their point of departure, their clinical experiences forge the essential waypoints along their path, knowledge and skills fuelling their navigation in rugged terrain towards their destination.

**Conclusions:**

Nursing students’ ethical competence evolved in intricate ways during their initial clinical period. Being informed bystanders or participants in the care of nursing home residents in situations of ethical tension may be a unique position enabling students to evaluate care options differently from those immersed in the ward culture. The findings indicate that organised professional development in nursing homes needs to focus on more reflexively driven ways of supervising students in their first clinical study period. Educational institutions need to continue and further develop reflection-based learning activities and meeting points with students and their peers during their clinical placement periods.

**Supplementary Information:**

The online version contains supplementary material available at 10.1186/s12912-024-02243-x.

## Background

Ethical competence is at the core of nurses’ integrity [1]. The overall aim of nursing is to deliver proficient healthcare services. However, the assessment of how services are delivered requires value-based and ethical inquiry [2]. Practicing nursing with integrity amid the complex moral choices and pressures that nurses confront on a daily basis is challenging [3]. Not living up to the ethical standards of nursing puts patients’ well-being at risk and is associated with human costs on patients’ behalf [4]. Furthermore, violating the ethical standards of nursing is also associated with moral injury [5]. To ensure high ethical standards in nursing, it is essential to enhance ethical training in the curriculum [6–8]; this training aims to cultivate moral qualities in students, preparing them for their roles as nurses. Being a ‘good’ nurse requires not only procedural skills and clinical reasoning but also strong moral qualities and ethical reasoning.

Improvements in nursing students’ ethical competence can positively affect appropriate and timely clinical outcomes [7]. The ways in which nursing and other healthcare professions educational institutions organise and implement learning activities to promote students’ ethical competence vary, and different approaches may yield diverse results [9–14]. However, the experiences students in the healthcare professions gain through their clinical studies substantial impact their learning outcome [15, 16]. According to Mezirow [17], encountering a real ethical problem initiates an inner process that is essential to the development of ethical competence. The process consists of two steps. First, one identifies the problem at hand. Then, the essence of the problem is mirrored in the self, and ethical reflection and assessment starts as an attempt to identify just solutions.

In Norway, although learning activities vary from institution to institution, a key requirement in the curriculum for a bachelor’s degree in nursing is that graduates are qualified to reflect on and handle ethical problems in their professional roles. Additionally, they must be qualified to plan and implement respectful, collaborative and comprehensive interactions with patients and the patients’ next of kin [18]. By their nature, these qualifications are related to clinical skills. Therefore, training and guidance to achieve mandatory qualifications cannot be separated from clinical studies, which make up 50% of the total study hours in nursing education in Norway.

In the bachelor’s programme in nursing at Oslo Metropolitan University [19] in Norway, nursing students’ ethical training during clinical studies emphasises developing ethical competence through a discursive model of ethical reflection. This model, which is known as the Center for Medical Ethics model, or the CME model [20], is extensively utilised across interdisciplinary settings in Norwegian health service settings. The application of ethical decision-making models is a widely used method for systematic training as a way to enhance ethically justified reasoning and well-grounded decision-making [21]. The CME model equips students with a practical and applicable tool for developing ethical competence. The model is considered to be well suited because of its relatively simple structure and ability to apply knowledge and assessment directly to subject matter and practical solutions. Furthermore, using the model enhances discourse and diversity in the structured process of addressing ethical and legal problems.

Over the years, researchers have conducted studies to better understand the concept of ethical competence in healthcare professionals. A review conducted by Kulju et al. [22] aimed to analyse the concept of ethical competence in the context of healthcare. Based on the results, ethical competence can be defined in terms of character strength, ethical awareness, moral judgement skills and willingness to do good. Virtuous professionals, the experience of professionals, human communication, ethical knowledge and supporting surroundings can be seen as prerequisites for healthcare personnel to develop ethical competence.

In an integrative review, Lechasseur et al. [23] aimed to clarify the concept of ethical competence, identifying six distinct yet interconnected components related to the concept: *ethical sensitivity*, the ability to recognise ethical tension; *ethical knowledge*, the integration of philosophical, normative, deontological and practical knowledge; *ethical reflection*, a continuous process evaluating alternatives to ethical issues; *ethical decision-making*, choosing responsibly among options; *ethical action*, contextually adapted action driven by reflection; and *ethical behaviour*, embodied moderation and respect. These components elucidate how ethical competence evolves among healthcare personnel and students.

Understanding the concept of ethical competence and its development in nursing students in different learning contexts is still challenging. The various studies addressing ethical competence in nursing students often target one of the six components identified by Lechasseur et al. [23], the relationship between two or more of the components or one of the components related to other phenomena. For example, Hakbilen et al. [24] found that students had medium ethical sensitivity after courses, highlighting the need to integrate ethical issues into all nursing education content to improve students’ ethical sensitivity. The results from a study conducted by Chen et al. [25] indicated that both moral sensitivity and professional values had a positive effect on the improvement of ethical decision-making in Chinese nursing students. Park et al. [26] reported that senior students had greater moral sensitivity in patient care and conflict than freshmen students and that more hours of ethics education were linked to higher principled thinking scores among seniors. The findings from another study [27] demonstrated that nursing students’ ethical sensitivity includes perception, affectivity, cognitive processing and cooperation. Using these categories, an educational framework for teaching ethical sensitivity was developed, detailing the purpose, content and methods. Albert et al. [28] conducted a review to understand nursing students’ ethical dilemmas in clinical settings; they found that dilemmas arose when students had to choose between providing ethical care or accepting unethical practices, staying silent about neglect or reporting it, and offering quality care or adapting to the culture because of a lack of autonomous decision-making. Heggestad et al. [29] explored the link between affective and cognitive empathy and students’ moral sensitivity, finding that affective empathy was a strong driver of students developing moral sensitivity. Students valued emotions for empathy and feared becoming indifferent. Moe [30] examined the relationship between ethical knowledge and action choices among graduating nursing students; she found a significant correlation between ethical knowledge and students’ actions after accounting for factors such as ethics courses, healthcare decision experience and general healthcare experience. ‘Action’ was measured by students’ likelihood of implementing behaviours in a list of nursing interventions across eight patient care vignettes. Ramos et al. [31] studied the ethical conflicts perceived by nursing students in their sixth semester and described their decision-making process. The students used a three-stage strategy: realisation, reflection and intervention. Reflection served as a mediator, drawing on individual insights, others’ input, academic knowledge and context, hence helping students choose the best response to ethical issues.

As the research literature has demonstrated, as a comprehensive concept, ethical competence has been underexplored. As a result, the concept has not yet been operationalised across different contexts and various healthcare settings, particularly in the context of first-year nursing students’ clinical practice in nursing homes. First-year nursing students often start their first period of clinical practice with limited formal ethical knowledge and experience; therefore, more knowledge of the evolution of ethical competence in them is necessary.

## Methods

### Aim of the study

The aim of the present study was to explore the development of ethical competence in first-year nursing students during their first clinical placements in nursing homes.

### Design

The current study employed an exploratory-descriptive qualitative (EDQ) design [32] and used content analysis to analyse the empirical data based on focus group interviews with students.

### Recruitment

All five researchers contributed to recruiting the participants. They provided information both face to face and via email to students during and after the clinical period. To be included, students had to (i) be enrolled in the academic year 2023–2024, (ii) have completed the clinical period at a nursing home, (iii) voluntarily participate in the study and (iv) agree to be recorded during the interviews. Students who wished to participate in the study contacted the researchers to arrange a date and time for the interviews.

### Participants and study setting

For the present study, purposive sampling was deemed appropriate for selecting participants who could provide the necessary information to address the study’s aim. Sandelowski [33, 34] suggested that, in qualitative research, it is crucial to ensure that the sample size is sufficient to obtain the quality of the information needed. A total of 113 first-year students were invited to participate in the study, and 28 agreed to participate. Among those who agreed, only three were men. The participants’ ages ranged from 19 to 31 years. At the time of the interviews, their work experience in healthcare services ranged from 0 to 8 years.

During the fall semester of 2023, the students attended a two-hour lecture and a three-hour seminar on ethics in nursing aimed at enhancing their theoretical ethical competence. The study was conducted at the Department of Nursing and Health Promotion, Oslo Metropolitan University, following the students’ completion of a six-week clinical period in a nursing home as part of their first-year curriculum during their bachelor’s degree in nursing.

### Data collection methods

The data were collected through focus group interviews using a semistructured interview guide. This data collection method is both time- and resource-efficient, allowing for the exploration of a wide variety of perceptions. Additionally, the participants can build upon each other’s responses, generating new insights and information [35]. The interview guide was developed for this study by the first author following the five phases recommended by Kallio et al. [36]: (1) identifying the prerequisites for using semistructured interviews; (2) retrieving and using previous knowledge; (3) formulating the preliminary semistructured interview guide; (4) pilot testing the guide; and (5) presenting the complete semistructured interview guide. After pilot testing, small adjustments to the questions were made to enhance the clarity and relevance of the interview guide. For the interview guide, see supplementary file 1.

Six focus groups were conducted between March and April 2024. The number of participants varied across the groups: two groups had three participants, two groups had five participants, and two groups had six participants. The interviews were held in a classroom or seminar room and lasted between 30 and 50 min.

### Ethical considerations

The present study was approved by the Norwegian Agency for Shared Services in Education and Research (SIKT, project number 334855). Approval to conduct the study was given by the head of studies at the Department of Nursing and Health Promotion at Oslo Metropolitan University. The study was conducted according to the World Medical Association Declaration of Helsinki’s [37] principles of good and ethical practices in scientific research: informed consent, consequences and confidentiality. All the students who participated provided written informed consent at the beginning of the interviews. The students were informed about the aim of the study and the data collection method and were guaranteed confidentiality and the ability to withdraw from the study at any point in time.

All the researchers moderating the focus groups were nurse educators engaged in students’ clinical studies in nursing homes, and hence some of the participants knew the moderator prior to data collection. Being invited to participate in a focus group conducted by a nurse educator could put students in vulnerable positions given that the nurse educator may be perceived as an authority figure. According to Barbour [35], researchers should carefully consider the reasons participants may have for taking part in a study. In this study, it would question morality if a student would resort to participate for fear of being disadvantaged or poorly assessed by the nurse educator. Furthermore, if a student would customize his or her statements in the focus group, to convey an image as a ‘good’ student in front of the nurse educator, it would question morality as well as data authenticity. Therefore, the researchers always strived to be as transparent and open as possible with participants regarding potential implications.

In this study, openness related to in-depth information to invited participants concerning the authenticity of willingness as a participating principle, as well as of the genuineness that any statements made in the focus group would not affect the student in any way. Furthermore, the students were detailed informed that the focus group would occur as a conversation with peers, the moderators’ role facilitating discussions, and that any viewpoints would be welcomed. Debriefing before and after the focus group sessions, as recommended by Barbour [35], disclosed no incidents of students feeling obliged to participate or feeling restrained to express themselves freely.

The interview data were stored as secured computer files that were accessible only to the researchers. The records and transcripts will be permanently deleted after the research project has been completed and the results have been published.

### Data analysis

All six focus group interviews were digitally recorded and transcribed verbatim by the researchers immediately after completion. The transcripts resulted in 52 A4 pages taped with 1.5 line spacing and New Roman font size. The duration from transcribing the interviews to completing the analysis ranged from four to six weeks.

To analyse the interviews, a phenomenological hermeneutical method inspired by Lindseth and Norberg [38] was used, which is ideal for understanding healthcare practices by exploring the narratives of lived experiences. This method involves three steps: naïve understanding, structural analysis and comprehensive understanding.

Initially, each researcher independently read the texts multiple times to gain a naïve understanding and highlighted passages describing nursing students’ perceptions of the preconditions that contributed to enabling their ethical competence. These passages were compared and discussed until a consensus was reached. The researchers then reread the texts to ensure that no relevant material was missed.

During the structural analysis, condensed meaning units were grouped into themes and reflected upon in light of the naïve understanding. The text was reassembled to integrate both naïve understanding and structural analysis. The literature was consulted to refine and deepen the understanding of the preconditions that contribute to enabling students’ ethical competence. A comprehensive understanding was developed, tested against the naïve understanding, discussed and revised multiple times, with further review given by the first author.

The data analysis concluded when that a comprehensive understanding of the meaning units was achieved, indicating saturation [39]. Coding meaning units involved reviewing each interview and noting every identified issue (or code). Saturation was reached when no new information emerged in any of the coded meanings [39].

### Rigour

Whittemore et al. [40] suggested four criteria to improve the rigour of a study with an EDQ design: credibility, authenticity, criticality and integrity.

To enhance study credibility, the researchers selected pertinent excerpts from participant statements to substantiate the findings. Researcher triangulation cross-verified the findings, enhancing credibility. Additionally, credibility was ensured through verbatim transcription of interviews and independent data analysis, which was followed by collaborative coding and theme development to reach a consensus.

Data authenticity was reinforced by encouraging the participants to freely express themselves during the interviews. The researchers supported and accurately represented participant voices in the findings.

The overall integrity of the study was evaluated through rigorous critical appraisal of the research decisions. A detailed description of the research process allows readers to comprehensively assess the study quality at every stage.

Criticality and integrity were further enhanced by addressing researcher bias, employing member checking and engaging in peer review. The researchers reflected on their positions relative to participants and maintained awareness of their preconceptions throughout. Reflexivity was pivotal in enhancing qualitative study rigour. Logistical challenges preclude member checking; however, the students were given ample opportunities during the focus group interviews to provide detailed input and clarify perspectives. Peer review involved all researchers critically evaluating the study for quality, validity and reliability. Although all team members reviewed the empirical data, only the first and last authors conducted structural analysis and developed a comprehensive understanding, with others providing expert assessments.

### Findings

The interpretation process and findings are derived from the transcripts of six focus group interviews. The findings are presented in three distinct parts: naïve understanding, structural analysis and comprehensive understanding.

### Naïve understanding

The nursing students, that is, the participants in the present study, encountered complex ethical situations during their clinical period in nursing homes, particularly when providing care to nursing home residents.

The students often encountered ethical challenges in clinical practice that conflicted with their theoretical knowledge of ethical care from university or from their personal values. They observed healthcare personnel engaging in behaviours they deemed unethical, such as ‘white lies’, causing feelings of powerlessness due to their inability to act. Despite witnessing challenging ethical caring actions, the students also noted positive examples of ethical sensitivity, actions and beneficiary caregiving. They navigated these complex situations by developing their own strategies and reflecting on their roles as future healthcare professionals.

The students recognised that their theoretical knowledge, practical skills and empathy for nursing home residents were crucial in identifying ethical problems. They internalised ethical principles deeply through their experiences, and this was often prompted by residents’ reactions. Although they emphasised the importance of guidance and reflective dialogue to enhance ethical competence, the lack of supervision and specific feedback on ethical problems was perceived as a hindrance to further developing ethical competence. The students valued discussions with peers, nurse educators and interdisciplinary meetings for support and ethical reflection, despite challenges in accessing consistent guidance from nurse preceptors.

### Structural analysis

The structural analysis of the data revealed three themes: (i) ethical competence forges in practice, (ii) ethical competence evolves at the intersection of knowledge and skills and (iii) ethical competence unfolds through meaningful discussions. These themes are considered essential preconditions for the development of ethical competence among nursing students. An example of the structural analysis process is illustrated in Table 1.

**Table 1:** Example of coding tree for theme (i)

Meaning units	Subtheme	Theme
You must take things as they come. You must observe body language. You must get to know them [residents] and try to navigate how to speak, which words to use (S1 FG1) In the clinic you see the small things between the lines (S1 FG2). Doing it, you understand reality, understand situations and what they entail (S4 FG4) You experience it with your body, you feel it, and then it settles more in you (S1 FG6)	Learning and experiencing being hands-on in real-word-situations	Ethical competence forges in practice
To groom [residents] is important. If it is not performed, it can lead to other problems (S1 FG1) It felt wrong to just leave the resident and let her starve. We had to do something (S4 FG5) We must keep motivating because it is important to maintain the residents’ resources (S1 FG5)	Being petitioned to act	
Some employees made fun of her [a resident] when she was angry. I thought it was crooked. Because even if we consider the things, she was angry about not to be real, her feelings indeed were real (S1 FG1) They [employees] held her hands, restricting her movement. It was done gentle, but still, it was coercion (S2 FG1). One comes a long way being person centered. I thought it was exciting to observe how those [nurses] who are experienced communicate. They avoid tension just by knowing how to communicate gentle and adapted (S3 FG1)	Discovering ethical and unethical care actions	
Imagining myself old and cognitive impaired, and how insecure I would feel if I was forced to do something. You see that they [residents] have anxiety, that they get scared, and that you make more harm forcing them than just to let it be (S1 FG1) I felt that the nurse in a way forced her action through. I think it would have been better that she [the resident] had her way (S3 FG1) I, myself, would have been more concerned asking the resident, “do you want to”, before doing things (S1 FG5) These are persons suffering from sicknesses, but they are human beings, and I think it breaks with what I consider “right” (S1 FG5)	Grounding the course of ones’ own actions	
What the patients says, makes me to flinch and pause (S3 FG2) If someone I care for should have been groomed in that manner or have done things to. One takes a step back and thinks of it … (S1 FG5)	Being triggered to reflect	

### Ethical competence forges in practice – being able to identify ethical and unethical care

This theme suggests that ethical competence is developed and strengthened through practical experience, with students having hands-on engagement in ethical situations and being petitioned to act. This implies that students can assess various situations and distinguish between ethical and unethical caregiving and start grounding the course of their own ethical actions through their reflections.

The clinical period was evaluated as a positive experience offering numerous valuable learning opportunities. The students observed healthcare personnel providing ethical and dignified care to nursing home residents. However, they also witnessed some situations in which the care provided was less ethical and undignified. The situations described by the students as unethical caregiving were situations in which the residents resisted care or various procedures, such as changing ostomy bags, or refused personal hygiene tasks, such as grooming or brushing their teeth. Intuitively, most students were aware of the conflict between their own wish to help and the residents’ autonomy and right to say ‘no’. The students observed that, in most situations, healthcare personnel respected the residents’ wish to refuse assistance and, therefore, did not provide any help. One of the students said the following:

In our facility, some residents had not washed themselves for a long time and smelled. However, I appreciated seeing how the principle of autonomy was maintained. I felt that the healthcare personnel managed this very well, ensuring that the residents made their own decisions. Nevertheless, I had concerns about hygiene. If a resident did not want to wash and the healthcare personnel respected their wish, I think that it raised an ethical dilemma … what about the principle of nonmaleficence and beneficence … how are these maintained? (S3, FG1)

Other situations identified as unethical practices and triggering students’ ethical reflection were those situations in which healthcare personnel, because of high workload, time pressure or ingrained habits, ignored residents’ needs and wishes and focused on getting the job done. One of the students said the following:

I think that’s one of the worst things. It truly irritates me when I see nurses doing a job, that they are doing it just because it’s a job. They don’t think about the fact that it’s a person who has had a whole life before they got here [at the nursing home]. I’ve experienced it so many times that for some nurses, it’s just about the job, just about completing tasks and getting it over with. … (S3, FG2).

### Ethical competence evolves at the intersection of knowledge and skills – Being able to apply theory in practice

This theme highlights the importance of both theoretical understanding (knowledge) and practical abilities (skills) in the development of students’ ethical competence. This finding suggests that ethical competence emerges as a result of the integration of knowledge and skills.

Several students felt that their ability to assess challenging situations as ethically laden resulted from their reflections on theoretical knowledge, practical skills and empathy for nursing home residents. Witnessing ethically challenging situations prompted them to reflect on ethical principles and find solutions, thereby acquiring and applying knowledge in practice. Students applied their knowledge, including their personal values and beliefs, as well as what they learned at university or during their clinical training, to assess the practical skills required for challenging situations. The skills the students often highlighted included the ability to initiate dialogue with residents to prevent ethical tensions, the capability to mediate and offer alternatives when residents did not comply with health measures, and the ability to articulate persuasive arguments to obtain residents’ consent for specific health measures they initially rejected but were deemed necessary.

Although the students were grateful for prior ethics education at the university and appreciated the knowledge gained from seminars, they believed that clinical practice provided real situations where they could reflect and discuss possible solutions. This is illustrated by one student’s statement:

When you’re in a nursing home, it’s much more real … when you discuss in the classroom, you won’t truly grasp the reality … yes, understanding the situation and what it entails … a case on paper is very black and white, but in reality, it involves emotions, observations and knowledge that help you assess the situation and find solutions. … (S4, FG4).

Nevertheless, the students perceived the nursing home as a valuable learning environment that offered many educational opportunities. Given that many nursing home residents had cognitive impairments or were persons with dementia, ethically challenging situations frequently arose. In addition to reflecting on how to apply their knowledge in practice, several students were interested in preventing the occurrence of ethical problems and/or finding solutions to these ethical situations. Therefore, the students stressed the importance of gaining skills using a model for ethical reflection, which helped them in ethical decision-making. This is illustrated by a student’s statement:

The CME model gave me a lot. I could organise things systematically and consider different courses of action. What can one choose to do and not do. In addition, then, I think in nursing homes, it’s special … you see so much, there are many residents who are diagnosed with dementia, and things where you stand in every day, such as assessing if they are competent to consent or not … Maybe you have to motivate a lot, spend a lot of time on diversion and such things … I think … it’s a place [nursing home] where you learn a lot about ethics. (S1, FG5)

### Ethical competence unfolds through meaningful discussions – providing opportunities to exchange knowledge

This theme emphasises the role of dialogue and reflection in the development of ethical competence. This suggests that engaging in meaningful discussions with peers, nurse preceptors or nurse educators facilitates the growth and deepening of ethical competence.

Although most of the students were content with the nursing home as a learning environment and the supervision offered by nurse preceptors, some students revealed feelings of dissatisfaction about observing healthcare personnel conducting unethical practices, including ‘white lies’, breaching of residents’ privacy, respect, removing personal items from the resident, such as cigarettes or a lighter, without residents’ consent or neglecting to display the medication when the resident requested. A situation involving unethical conduct was illustrated by one of the students:

One resident was very sad and anxious about being there [nursing home]. She asked several times, ‘When will I get to go home?’ And then the nurse preceptor said, among other things, that ‘you are going home in three days’. However, that’s not true … The patient was on a two-week stay. The nurse preceptor said she did this to calm down the resident’s anxiety; she gave the resident hope … We discussed a little bit … and I think that it’s a bit of an ethical dilemma because the nurse said this to be kind, but she [the resident] had the right to know about her stay. (S1, FG6)

In such situations, the students felt the urge to discuss their emotions and their own assessments with someone. However, the nurse preceptors were often too busy, restricting their time for reflection and thorough supervision. As a result, the students turned to their peers or nurse educators for discussions. Often students felt safer discussing with peers and educators and found these discussions more helpful. If these discussions were supportive, they could lead the students to find confidence in their assessments and could help them find their voice, advocating for the residents and for ethical behaviour towards the residents.

However, the students also displayed positive experiences. Observations of commendable ethical actions and behaviour from staff and nurse preceptors demonstrating effective techniques for handling value-laden situations helped the students recognise and integrate beneficial ethical actions and behaviours into their own repertoires. At times, both students and healthcare personnel from the same ward were regularly provided with opportunities to reflect on their practices, particularly ethically challenging situations. These group discussions were facilitated by a priest. One student expressed enthusiasm for these discussions:

We had a visit from the priest in the ward to discuss ethical challenges. We stood in a circle, and the healthcare personnel shared the ethical challenges they faced in their daily work. We discussed various ethical dilemmas and explored the best ways to address them so that everyone involved would benefit. I found it quite rewarding to engage in these discussions. Although we did not reach a definitive conclusion, I felt that having such discussions improved my ethical knowledge and understanding. (S3, FG3)

### Comprehensive understanding

The naïve understanding and structural analysis illustrate that nursing students embark on a journey towards ethical competence during their first period of clinical studies, with clinical experiences being essential waypoints along the path to their destination, that is, gaining ethical competence. Ethical sensitivity is foundational and the point of departure for the evolution of ethical competence in students. This sensitivity is driven by the compassion and understanding of residents’ needs and best interests, which students observe firsthand. Ethical knowledge evolves at the intersection of theoretical and practical insights within the nursing home setting and fuels and propels students’ ability to assess and reflect upon options in situations where there is ethical tension. Making meaningful discussions with their fellow passengers; peers, nurse preceptors and educators are crucial preconditions fostering students’ ethical reflections and decision-making in navigating the clinically rugged terrain. Ethical action and behaviour are the destinations of students’ journeys and hinge upon their learning of beneficial caring skills and on their opportunities to behave in accordance with their values in their caregiving. Overall, nursing students’ ethical competence evolves when they integrate ethical sensitivity, knowledge, reflection, decision-making, action and behaviour within clinical practice.

## Discussion

First-year nursing students with limited knowledge and skills enter their initial clinical studies and likely do not achieve the final level of ethical competence required in the nursing profession during this period. Nevertheless, our comprehensive understanding is that first-year nursing students begin a crucial journey towards ethical competence during their clinical placement in nursing homes. Their journey progresses through the integration of various components of ethical competence. Most prominently, the characteristics of ethical competence described in the integrative review of Lechasseur et al. [23] are identifiable in the scenic route the students’ take. Each of the components of ethical competence defined by Lechasseur et al. [23] evolve in the students, both distinctly and intertwined. Therefore, we find it valuable to discuss the students’ roadmap considering the components of ethical competence outlined by Lechasseur et al. [23]. We will discuss how ethical sensitivity, ethical knowledge, ethical reflection, ethical action and ethical behaviour play out in our finding, offering interpretations of how ethical competence evolves throughout their journey.

### Ethical sensitivity is the point of departure

In all six focus groups, the students provided rich descriptions of the ethical problems that they encountered in their clinical studies and discussed how they individually identified ethical tension in these situations. Drawing on Weaver et al. [41], Lechasseur et al. [23] define ethical sensitivity in nursing as both a compassionate-driven and intelligence-driven capacity. The compassionate aspect is related to self-awareness and personal normative knowledge and is particularly described by the students in our study as to ‘imagine themselves in the role of the resident’ as a catalyst for them to identify tension.

The students were strongly affected by the residents’ vulnerability in value-laden situations when they sensed that the residents were not given choices and had to accept receiving care they did not consent to, involuntarily had to relate to strangers, felt that their intimate spheres were being invaded, felt that customised information was not given to them and so forth. For example, one student described her emotional discomfort witnessing a residents’ vulnerability being restricted access to his personal items (cigarettes and lighter). This caused the resident to be restless and his hands to shake.

The students’ emotions appear as a point of departure in their journey towards ethical competence enabling them to identify tension. In this sense, our findings are in line with those of Heggestad et al. [29], who found that undergraduate nursing students’ moral sensitivity is mostly linked to the affective dimension of empathy.

Studying senior nursing students, Shayestehfard [27] found that both affective and cognitive dimensions characterise ethical sensitivity in nursing students. In their study, nursing students’ ethical sensitivity is described as being affected emotionally and, hence, becoming aware, awakened, alarmed or shaken. The cognitive side to students’ sensitivity is described as processing signs and symptoms of vulnerability that enable the students to identify caring needs. The students in our study also described the cognitive components of their ethical sensitivity. Many of the descriptions in the focus group discussions represented their interpretations of the consequences of not intervening, professional caring duties in nursing and the beneficence or nonmaleficence of residents in critical situations as strong sources of their identification of ethical tension. Some of the students expressed that they were initially emotionally driven in their identification of ethical problems. However, as time progressed, their understanding and focus could change. One student related this change directly to the forging of ethical competence. As competence forges, the student said, one understands better what is ethically problematic and what is not, and you are not only driven by your feelings, but you are also driven by what you understand to be critical caring needs.

### Ethical knowledge – pit stopping to fuel

Sometimes, the students identified ethical tension merely depending on their inherent and personal normative knowledge and values, but often, they also applied other knowledge, especially in the longer term. Lechasseur et al. [23] define ethical knowledge as multifaceted, including philosophical and theoretical knowledge as well as practical knowledge.

Ethical sensitivity is sometimes solely normative driven, but it is difficult to negate the relevance of factual knowledge in ethical reflection and decision-making [31, 42]. Few would deny the importance of factual knowledge concerning the assessment of treatment and healthcare, consequences and obligations for specific patients or their next of kin that lead to decisions [42]. Thus, philosophical, and theoretical knowledge (‘*knowing that*’) is a necessary condition for assessing options and making decisions. The students described knowledge from medicine, nursing, psychology, and other disciplines as sources of ethical reflection, most frequently concerning the doctrine of informed consent, values and care needs at stake, ethical principles, ethical guidelines or directives and communication strategies and interaction. One student said that theoretical knowledge learned at university prior to clinical studies was ‘worth its weight in gold’. Nevertheless, philosophical, and theoretical knowledge is not merely something one learns and can apply instantly [7, 43, 44]. Decontextualized ethics education does not sufficiently help students transfer learning from the classroom to ethical nursing practice situations [45]. During the focus group interviews, the students highlighted the importance of the nursing home as a learning arena contributing to their acquisition of ethical knowledge in the form of practical skills. This means that the clinical studies offered them a real-world framework consisting of authentic situations, hence enabling students to acquire practical knowledge (‘*knowing how*’) through a meaningful understanding of the context at hand. Therefore, the students’ journey towards ethical competence hinges on understanding the particularity of each ethical problem, such as what and who it concerns in the specific context in which the problem arises and unfolds. These understandings fuel the forging of ethical competence among students.

Sometimes, the students described situations in which they experienced a noticeable lack of knowledge, which led them to question which skills were applicable and appropriate. According to Andersson et al. [9], to become ethically competent in clinical studies, three moments should be presented to students: the learning environment should create conditions for learning, nurse preceptors should design strategies for learning, and students should interact with others. The present study has revealed how the students described the nursing home as a learning environment in which encounters are both physical and personal; they learned to recognise and interpret residents’ verbal and nonverbal language and its meanings, and they were required to address ethical problems by applying practical skills and understanding. Concrete situations often triggered their inquiries. In their search for practical knowledge, the students had to interact with others, which fuelled and facilitated their journey towards achieving ethical competence.

### Ethical reflection and ethical decision-making – navigating rugged terrain

Clinical studies provided the nursing students with the opportunities to engage in ethical reflection and decision-making processes in their placement wards. The students frequently reflected on the actions to take in various ethically tense situations and often provided sound justifications for their suggestions or raised relevant questions about what to consider. A recurring theme in their discussions was how much to push residents who resisted healthcare, speculating on the least invasive approaches. Ethical reflection represents the thinking process helping decision makers clarify beliefs and thoughts by considering various alternatives to ethical problems [23]. Ethical decision-making is closely intertwined with the reflective process, but differs in the sense that, when ‘deciding’, one also makes a choice from among the number of alternatives and thereby temporarily pauses the reflective process [23]. Nevertheless, being a student, especially encountering the clinical field for the first time, is a susceptible position, and it might be challenging for students to reflect on and make decisions. Particularly during the initial period of their placement, students mostly take part in caring actions that are already relatively settled and must learn how to implement prescribed caring chores.

Ranjbar et al. [46] developed a three-level model illustrating the route for moral development in nursing students. To achieve the final level of ethical competence, the students first pass through phases of development at the lower levels. As students enter their first year and are exposed to clinical practice, they begin acquiring practical and technical skills (first level). Here, their focus is essentially on acquiring and mastering these skills and being able to perform them with ease. It is at the second level that students advance in their ethical judgements. At level two, students can better reconstruct ethical problems and become more effective ethical agents. Until students reach level two, it is difficult for them to make sound ethical assessments without the support of others. In our study, the students’ abilities to take part in ethical reflections and ethical decision-making in practice varied. Some students were not very active in discussions concerning alternative actions and interventions to resolve ethical problems in their ward, but they expressed that they learned from observing and listening. Other students displayed a much more active role in the decision-making processes, discussing and sharing their views and opinions with staff, hence influencing the actions taken. Furthermore, the students often encountered ethical problems that arose unexpectedly or spontaneously and sometimes had to reflect there-and-then and decide what to do, often without supervision and without a prescribed recipe.

However, the process of learning how to understand and make decisions when encountering ethical problems in real-life nursing contexts continues to progress throughout the bachelor’s programme [21]. Managing ethical problems gave the students and participants confidence, triggering their potential to solve ethical problems now and in the future. However, the students expressed a need to take part in discussions and reflect on how to go forward in situations with ethical tension because they lacked the experience, knowledge and skills to navigate through ‘rugged terrain’. In many cases, the students said that, in their opinion, communication is crucial in solving ethical problems, and they need to reflect on and increase their skills using viable techniques and manners to initiate dialogue, mediation and persuading arguments.

According to Jakobsen et al. [21], using group discussions and discussing examples of ethical problems can help students engage in a more comprehensive reflection process. Discourse within the clinical setting has been outlined as a prerequisite concerning ethical competence [31, 42]. Justifying a certain practice requires more than the competence of one individual; hence, the decision-making process should be based on a multitude of views and opinions, thus contributing to consolidating decisions throughout the healthcare team [42]. Crucial elements of knowledge and skills are transferred among clinicians through their mutual reflections on appropriate care actions. Hence, being part of ethical reflection is essential to nursing students’ learning [31]. According to a review of the empirical literature on nursing students’ ethical decision-making, discussions with contemporaries are perceived as the most influential source of developing ethical decision-making abilities [47]. In their study, Ramos et al. [31] found that ethical reflection serves as a mediator between the realisation of an ethical problem and the interventions that nursing students carry out. Discussing the problem at hand helps nursing students draw on both individual insights and others’ input, helping the students choose the best response to ethical issues. Sometimes, when lacking the opportunity to reflect, the students turned to peers and educators as sparring pairs, pondering the alternatives to problems. The students found peers to be central in this regard, with peers being in circumstances similar to themselves. Reflecting together with another person was essential to their understanding and ability to develop skills for assessing and knowing how to handle difficult situations. Using the CME model [20] was considered a fruitful method of sorting information as well as sorting objective facts from subjective emotions, hence resulting in greater clarity.

The students valued ethical reflection partly because they believed that some healthcare personnel’s interventions were based on poor assessments that did not sufficiently advocate for the residents; they also found that nurse preceptors or other employees were sometimes more concerned with getting the job done, avoiding extra work, and coping through the working day when assessing solutions to ethical problems. The students often mentioned ‘reflection’ both as a way of considering alternatives of action when facing ethical problems and as a way of processing their emotions while witnessing poor care and not having the courage to question nurse preceptors’ assessments. The results from a literature review on moral courage in undergraduate nursing students [48] highlight students’ strong identification with the role of patient advocacy. The same attitude was perceived by the participants in our study; they expressed that residents’ well-being was their primary objective. However, according to Bickhoff et al. [48], several factors inhibit nursing students from pursuing an advocacy role, sometimes also leading to students taking part in poor practice unintentionally or complying with unethical practices. The characteristics of nursing students’ experiences in their clinical studies, such as feeling or being subordinate on the ward, their identity as ‘just’ or ‘only’ a student, a desire to fit in, fear of reprisals and poor validation, have been reported in numerous studies [48–51]. These experiences diminish nursing students’ opportunities to take active roles in ethical reflections and decision-making and, thus, to take part in and influence the ward learning community. On the other hand, students who are given opportunities or who seize opportunities on their own to act as patients’ advocates report gaining the courage to voice their assessments and suggestions in the work environment [48]. The participants in our study expressed that they sometimes felt subordinate to the ward and were not given a voice, which hindered their ability to make sound assessments for the benefit of the residents. Nevertheless, some students, though not all, found the ability to seize opportunities to advocate for residents’ beneficial care.

### Ethical action and ethical behaviour – the destination in sight

Ethical action involves implementing a course of action chosen after considering the possible alternatives, while ethical behaviour is characterised by an attitude of respect, responsiveness and support when carrying out the chosen action [23]. In a person with high ethical competence, ethical behaviour is embodied [23]. The students discussed actions they carried out to solve ethical problems, sometimes with nurse preceptors, peers or independently. Similar to the results of a systematic review [7], our study has revealed that students prioritised maintaining patients’ dignity, privacy and confidentiality, emphasised effective communication to resolve ethical issues and demonstrated a strong commitment to residents’ well-being. Consequently, the students showcased sound ethical behaviour.

Some students described the inability to act in accordance with ethical standards as a bodily experience of distress. One student mentioned that her feelings in tense situations settled in her body. According to Ranjbar et al. [46], the final level (level three) in the route for moral development in nursing students is where they internalise professional values and the ethos of nursing into their personal identity, making ethical actions and behaviour a reflection of their identity and personality. Few students reach this level during their nursing education [46]. Although it is difficult and impractical for us to claim that the students in our study had reached level three, their discussions rarely involved knowingly violating ethical values. When they did, they expressed discomfort. For example, one student described lying to a resident to get him indoors when he was not properly dressed because of cold weather, finding the experience distressing. Similar findings have also been presented in previous studies [52, 53]. Nevertheless, even though nursing students tend to be idealistic and hold strong values [7], there is no guarantee that these values will consistently result in virtuous ethical actions and behaviours, either now or in the future.

A literature review investigating nurses’ ethical practices [54] revealed that contextual circumstances, such as limited time and resources and less authority in the ward hierarchy, can influence nurses’ ability to make decisions. These circumstances result in a conformist approach, adapting to existing practices and sometimes merely executing the decisions made by others. This, in turn, affects nurses’ internalisation of ethical behaviour and leads to less individually adapted care actions. Although this review [54] focused on professional nurses, there is evidence that nursing students’ ethical behaviour is similarly influenced by these circumstances. For example, Tanaka [55] reported that nursing students often adjust their ethical values to accommodate certain circumstances. Even when nursing students perceive the actions and behaviours of nurse preceptors as ‘wrong’, they may alter their stance on the issue and reorganise their ethical values to adopt coping behaviours that align with the situation. As a result, nursing students often struggle with prioritising their relationships, with nurses who exhibit inadequate care behaviours or with their commitment to patients. As nursing students progress through their programmes, they may become increasingly disillusioned, cynical and focused on completing their tasks, which ultimately ends with a loss of idealism [56].

The students participating in our study described instances in which they observed cynicism among certain employees and witnessed violations of residents’ dignity by nurse preceptors and other healthcare personnel. Examples included speaking over residents’ heads, ridiculing them and handling them roughly or even being disrespectful to residents’ family members. In these situations, the students often felt powerless and complicit, feeling ‘dragged along’ because of their subordinate role. Occasionally, they sought explanations from the nurse preceptors, but the responses varied in credibility. The students also observed commendable ethical actions from nurse preceptors, who demonstrated effective techniques for handling value-laden situations. Learning from these positive examples helped the students recognise ethical behaviours. They observed that the manner of coercion mattered and witnessed gentle, informative restraint techniques. Overall, the students were eager to adopt noncoercive approaches, seeking to balance assertiveness with positive persuasion.

Despite witnessing unethical actions and behaviours, these experiences did not necessarily hinder the students’ development of ethical competence. According to Engel et al. [57], experiencing unethical caregiving can actually strengthen students’ commitment to ethical practices. Most students act as informed bystanders and can navigate the space between observation and action. They may maintain a low profile while also developing the courage to advocate for patients. These adaptive strategies were evident among the students in our study. In distressing situations, the students occasionally assumed caregiving responsibilities to ensure proper care. For instance, when an employee force-fed a resident, a student intervened by gently and patiently helping the resident. We interpret this action as a coping strategy that bridges the gap between observation and action.

The students frequently mentioned that gaining confidence during their clinical studies might empower them to voice their concerns more effectively in the future. However, according to Bickhoff et al. [48], the lack of authority persists throughout the clinical curriculum, with students often assuming a subordinate role across different periods of clinical study. On a positive note, some students have reported that experiencing a lack of authority motivates them to avoid unethical practices in their future careers [48]. In our study, the students strongly expressed their desire to maintain their ability to advocate for residents’ best interests in the future. They observed that, for some healthcare personnel, the job had become routine, leading to a loss of perspective on ethical actions. The students were determined not to find themselves in a similar position in their careers.

### Implications for nursing education and clinical practice

Findings from the current study suggest that building a solid ethical foundation in nursing involves more than just theoretical and practical knowledge. Several strategies are needed to sustain ethical practice and potentially mitigate moral distress among nursing students and nurses.

One crucial strategy is to integrate ethics education throughout the entire curriculum, rather than treating it as a standalone module. This should include case studies, role-playing, and discussions relevant to real-world scenarios. Additionally, providing strong mentorship and supervision during clinical placements is vital. Experienced nurse educators and preceptors can model ethical behavior, offer guidance, and support students as they navigate complex situations. Encouraging regular reflective practice is also important. Discussions, debriefing sessions, and peer interactions can help students process their experiences and deepen their understanding of their ethical beliefs and responses. Since learning does not occur in a vacuum, creating a supportive learning environment with access to guidance can assist students and nurses in addressing moral distress and seeking guidance. Furthermore, offering diverse and challenging experiential learning opportunities allows students to confront ethical issues in a controlled setting, hence building their confidence and competence in managing ethical problems. Finally, fostering a culture of ethical leadership within educational and clinical environments can set a standard of ethical behavior that positively influences nursing students and nurses.

### Strengths and limitations of the study

The present study’s findings are based on participating students’ accounts of their experiences with ethical issues during their first clinical period in nursing homes. One limitation of the study is that the students’ subjective perceptions and narratives may not always accurately reflect their actual ethical actions and behaviour in a real-world caring context.

Out of the 113 invited students, only 28 agreed to participate. It is worth considering whether those students who chose not to participate would have described their experiences differently. Although the sample size was limited to 28 participants across six focus groups, our transcription and analysis of the data indicated that saturation was achieved after the final focus group. Furthermore, our goal has not been to generalise but rather to present a possible interpretation of how ethical competence evolves in nursing students in the context of clinical studies in nursing homes.

Another potential limitation could stem from our interpretations, which might be shaped by our preconceptions, particularly because all the authors are nurse educators engaged in students’ clinical studies in nursing homes and know the curriculum well. To counter potential bias, we rigorously adhered to the data as they emerged from the transcripts.

Another challenge we encountered relates to the varied use of concepts concerning ethical practices by different authors [23, 54]. While reviewing the literature on nursing students and their ethical competence, we observed that some studies aimed at exploring or describing ethical decision-making in students but primarily focused on aspects of ethical sensitivity. Therefore, in certain instances, we needed to heavily rely on specific findings from these studies, even if they did not align perfectly with their original aims. Throughout this process, we aimed to maintain accuracy and fairness in referencing other studies.

Some of the studies we have referenced in our discussion were older than five years and were conducted in contexts that may not resemble the environment of Norwegian or Nordic nursing education. Because of the limited research exploring ethical competence in nursing students, we reviewed these studies to provide supporting knowledge for our findings. The geographical differences between the places where the studies were conducted and our study could have led to discrepancies in our discussion of the development of students’ ethical competence during their initial clinical study period in Norwegian nursing homes. However, we did not find studies with aims similar to ours, so our discussion draws on the findings of Lechasseur et al. [23] and the general literature describing nursing students’ experiences with ethical issues in diverse and varying contexts. However, we believe that ethical competence in nursing students evolves during their theoretical and clinical education, regardless of their country of origin; therefore, our findings could be applicable to similar settings and practices among nursing students.

## Conclusion

The aim of the present study was to explore the development of ethical competence in first-year nursing students during their first clinical placements in nursing homes. Our naïve understanding was that the ethical challenges students encounter in their clinical studies often conflict with their theoretical ethical knowledge and personal values. The students navigated complex ethical situations by reflecting on their experiences, expanding and adapting their knowledge base and internalising ethical principles in their actions. Through structural analysis, we identified three key themes that are considered essential preconditions for the development of ethical competence in nursing students. Our comprehensive understanding of the findings reveals that nursing students embark on a journey towards ethical competence, with clinical experiences serving as pivotal milestones.

Our findings illustrate that nursing students’ ethical competence evolves in complex ways when they first time are exposed to clinical settings. The first clinical period in nursing homes offers real-life insights into both ethical and less ethical care practices. Acting as informed observers or active participants in ethical problems involving nursing home residents offers students a unique perspective to critically evaluate care options that are distinct from those immersed in the ward culture. However, students’ ability to resist conventional practices and progress on their ethical journey may vary. Moments of reflection and supportive interactions are crucial for sustaining momentum on this journey. Therefore, peers, nurse preceptors, and nurse educators play vital roles in guiding students by providing robust support and navigation.

Our study suggests that structured professional development in nursing homes should prioritise the reflective supervision of students during their initial clinical placements. Educational institutions should continue to enhance and expand ethical reflection-based learning activities and opportunities for students and their peers during their clinical training.

### Electronic supplementary material

Below is the link to the electronic supplementary material.

Supplementary Material 1

## Data Availability

The datasets used and/or analyzed during the current study are available from the corresponding author upon reasonable request.

## Abbreviations

S: Student [number] participant in focus group interviews
FG: Focus group [number]

## References

[1] Hemberg J, Hemberg H. Ethical competence in a profession: Healthcare professionals’ views. Nurs Open. 2020;7(4):1249–59. 32587745 10.1002/nop2.501 PMC7308671

[2] Kangasniemi M, Pakkanen P, Korhonen A. Professional ethics in nursing: an integrative review. J Adv Nurs. 2015;71(8):1744–57. 25598048 10.1111/jan.12619

[3] Ulrich CM, Taylor C, Soeken K, O’Donnell P, Farrar A, Danis M, Grady C. Everyday ethics: ethical issues and stress in nursing practice. J Adv Nurs. 2010;66(11):2510–9. 20735502 10.1111/j.1365-2648.2010.05425.x PMC3865804

[4] Waterfield D, Barnason S. The integration of care ethics and nursing workload: a qualitative systematic review. J Nurs Manag. 2022;30(7):2194–206. 35704019 10.1111/jonm.13723 PMC10084060

[5] Čartolovni A, Stolt M, Scott PA, Suhonen R. Moral injury in healthcare professionals: a scoping review and discussion. Nurs Ethics. 2021;28(5):590–602. 33427020 10.1177/0969733020966776 PMC8366182

[6] Robichaux C. Developing ethical skills: from sensitivity to action. Crit Care Nurse. 2012;32(2):65–72. 22467614 10.4037/ccn2012929

[7] Parandeh A, Khaghanizade M, Mohammadi E, Mokhtari Nouri J. Factors influencing development of professional values among nursing students and instructors: a systematic review. Glob J Health Sci. 2014;7(2):284–93. 25716397 10.5539/gjhs.v7n2p284 PMC4796667

[8] Standing M. Clinical decision-making skills on the developmental journey from student to registered nurse: a longitudinal inquiry. J Adv Nurs. 2007;60(3):257–69. 17850295 10.1111/j.1365-2648.2007.04407.x

[9] Andersson H, Svensson A, Frank C, Rantala A, Holmberg M, Bremer A. Ethics education to support ethical competence learning in healthcare: an integrative systematic review. BMC Med Ethics. 2022;23(1):29. 35305627 10.1186/s12910-022-00766-z PMC8933936

[10] Cannaerts N, Gastmans C, Dierckx de Casterlé B. Contribution of ethics education to the ethical competence of nursing students: educators’ and students’ perceptions. Nurs Ethics. 2014;21(8):861–78. 24714046 10.1177/0969733014523166

[11] Khatiban M, Falahan SN, Amini R, Farahanchi A, Soltanian A. Lecture-based versus problem-based learning in ethics education among nursing students. Nurs Ethics. 2019;26(6):1753–64. 29716419 10.1177/0969733018767246

[12] Kaşıkçı M, Yıldırım Z. Interventions to improve ethical decision-making skills in nursing students: a systematic review. Nurs Ethics 2024:9697330241239917. 10.1177/09697330241239917 38576333

[13] Ozgonul L, Alimoglu MK. Comparison of lecture and team-based learning in medical ethics education. Nurs Ethics. 2019;26(3):903–13. 28946799 10.1177/0969733017731916

[14] Zhang F, Zhao L, Zeng Y, Xu K, Wen X. A comparison of inquiry-oriented teaching and lecture-based approach in nursing ethics education. Nurse Educ Today. 2019;79:86–91. 31108384 10.1016/j.nedt.2019.05.006

[15] Pitkänen S, Kääriäinen M, Oikarainen A, Tuomikoski AM, Elo S, Ruotsalainen H, Saarikoski M, Kärsämänoja T, Mikkonen K. Healthcare students’ evaluation of the clinical learning environment and supervision - a cross-sectional study. Nurse Educ Today. 2018;62:143–9. 29353088 10.1016/j.nedt.2018.01.005

[16] Vizcaya-Moreno MF, Pérez-Cañaveras RM, Jiménez-Ruiz I, Herrero JJ. Student nurse perceptions of supervision and clinical learning environment: a phenomenological research study. Enferm Glob. 2018;17(3):319–31. 10.6018/eglobal.17.3.276101

[17] Mezirow J. Transforamtive dimensions of adult learning. San Francisco: Josey-Bass; 1991.

[18] New Regulation on. a common framework for health-and social studies [In Norwegian]. Retrieved 05.05.2024 from: https://lovdata.no/dokument/SF/forskrift/2017-09-06-1353

[19] Oslo Metropolitan University: Bachelor’s Programme in Nursing. Oslo: Department of Nursing and Health Promotion. 2023. Retrived 07.05.2024 from: https://student.oslomet.no/en/studier/-/studieinfo/programplan/SYKP/2023/H%C3%98ST

[20] Førde R, Pedersen R. Clinical ethics committees in Norway: what do they do, and does it make a difference? Camb Q Healthc Ethics. 2011;20(3):389–95. 21676326 10.1017/S0963180111000077

[21] Jakobsen LM, Sunde Maehre K. Can a structured model of ethical reflection be used to teach ethics to nursing students? An approach to teaching nursing students a tool for systematic ethical reflection. Nurs Open. 2023;10(2):721–9. 36097342 10.1002/nop2.1339 PMC9834543

[22] Kulju K, Stolt M, Suhonen R, Leino-Kilpi H. Ethical competence: a concept analysis. Nurs Ethics. 2016;23(4):401–12. 25670176 10.1177/0969733014567025

[23] Lechasseur K, Caux C, Dollé S, Legault A. Ethical competence: an integrative review. Nurs Ethics. 2018;25(6):694–706. 27694548 10.1177/0969733016667773

[24] Hakbilen HG, Ince S, Ozgonul ML. Ethical sensitivity of nursing students during a 4-Year nursing curriculum in Turkey. J Acad Ethics. 2023;21(1):41–51. 10.1007/s10805-021-09432-2

[25] Chen Q, Su X, Liu S, Miao K, Fang H. The relationship between moral sensitivity and professional values and ethical decision-making in nursing students. Nurse Educ Today. 2021;105:105056. 34265538 10.1016/j.nedt.2021.105056

[26] Park M, Kjervik D, Crandell J, Oermann MH. The relationship of ethics education to moral sensitivity and moral reasoning skills of nursing students. Nurs Ethics. 2012;19(4):568–80. 22691600 10.1177/0969733011433922

[27] Shayestehfard M, Torabizadeh C, Gholamzadeh S, Ebadi A. Ethical sensitivity in nursing students: developing a context-based Education. Electron J Gen Med. 2020;17(2):e195. 10.29333/ejgm/7812

[28] Albert JS, Younas A, Sana S. Nursing students’ ethical dilemmas regarding patient care: an integrative review. Nurse Educ Today. 2020;88:104389. 32193068 10.1016/j.nedt.2020.104389

[29] Heggestad AKT, Nortvedt P, Christiansen B, Konow-Lund AS. Undergraduate nursing students’ ability to empathize: a qualitative study. Nurs Ethics. 2018;25(6):786–95. 27605557 10.1177/0969733016664982

[30] Moe CS. Relationship of ethical knowledge to action in senior baccalaureate nursing students. Nurs Educ Perspect. 2018;39(6):363–5. 29621033 10.1097/01.NEP.0000000000000319

[31] Ramos FR, Brehmer LC, Vargas MA, Trombetta AP, Silveira LR, Drago L. Ethical conflicts and the process of reflection in undergraduate nursing students in Brazil. Nurs Ethics. 2015;22(4):428–39. 25096246 10.1177/0969733014538890

[32] Hunter D, McCallum J, Howes D. Defining exploratory-descriptive qualitative (EDQ) research and considering its application to healthcare. J Nur Healthc. 2019;4(1):1–8.

[33] Sandelowski M. Whatever happened to qualitative description? Res Nurs Health. 2000;23(4):334–40. 10940958 10.1002/1098-240X(200008)23:4<334::AID-NUR9>3.0.CO;2-G

[34] Sandelowski M. What’s in a name? Qualitative description revisited. Res Nurs Health. 2010;33(1):77–84. 20014004 10.1002/nur.20362

[35] Barbour R. Doing focus groups. In., 2nd edn. Edited by Flick U. Los Angeles: SAGE Publications; 2018.

[36] Kallio H, Pietilä A-M, Johnson M, Kangasniemi M. Systematic methodological review: developing a framework for a qualitative semi-structured interview guide. J Adv Nurs. 2016;72(12):2954–65. 27221824 10.1111/jan.13031

[37] WMA. World Medical Association Declaration of Helsinki: ethical principles for Medical Research Involving human subjects. JAMA. 2013;310(20):2191–4. 24141714 10.1001/jama.2013.281053

[38] Lindseth A, Norberg A. A phenomenological hermeneutical method for researching lived experience. Scand J Caring Sci. 2004;18(2):145–53. 15147477 10.1111/j.1471-6712.2004.00258.x

[39] Hennink M, Kaiser BN. Sample sizes for saturation in qualitative research: a systematic review of empirical tests. Soc Sci Med. 2022;292:114523. 34785096 10.1016/j.socscimed.2021.114523

[40] Whittemore R, Chase SK, Mandle CL. Validity in qualitative research. Qual Health Res. 2001;11(4):522–37. 11521609 10.1177/104973201129119299

[41] Weaver K, Morse J, Mitcham C. Ethical sensitivity in professional practice: concept analysis. J Adv Nurs. 2008;62(5):607–18. 18355227 10.1111/j.1365-2648.2008.04625.x

[42] Reiter-Theil S, Mertz M, Schürmann J, Stingelin Giles N, Meyer-Zehnder B. Evidence - competence - discourse: the theoretical framework of the multi-centre clinical ethics support project METAP. Bioethics. 2011;25(7):403–12. 21790694 10.1111/j.1467-8519.2011.01915.x

[43] Fantinelli S, Cortini M, Di Fiore T, Iervese S, Galanti T. Bridging the gap between theoretical learning and practical application: a qualitative study in the Italian Educational Context. Educ Sci. 2024;14:198. 10.3390/educsci14020198

[44] Hatlevik I, Smeby J-C. Programme coherence and epistemological beliefs. Nordic Psychol. 2015;67(2):136–53. 10.1080/19012276.2015.1031553

[45] Krautscheid L, Brown M. Microethical decision making among baccalaureate nursing students: a qualitative investigation. J Nurs Educ. 2014;53(3):S19–25. 24512334 10.3928/01484834-20140211-05

[46] Ranjbar H, Joolaee S, Vedadhir A, Abbasszadeh A, Bernstein C. An Evolutionary Route for the Moral development of nursing students: a Constructivist grounded theory. J Nurs Res. 2018;26(3):158–67. 28858973 10.1097/jnr.0000000000000224

[47] Numminen OH, Leino-Kilpi H. Nursing students’ ethical decision-making: a review of the literature. Nurse Educ Today. 2007;27(7):796–807. 17166636 10.1016/j.nedt.2006.10.013

[48] Bickhoff L, Sinclair PM, Levett-Jones T. Moral courage in undergraduate nursing students: a literature review. Collegian. 2017;24(1):71–83. 29218965 10.1016/j.colegn.2015.08.002

[49] Bril I, Boer HJ, Degens N, Fleer J. Nursing students’ experiences with clinical placement as a learning environment for assertiveness: a qualitative interview study. Teach Learn Nurs. 2022;17(4):383–91. 10.1016/j.teln.2022.04.006

[50] Najafi Kalyani M, Jamshidi N, Molazem Z, Torabizadeh C, Sharif F. How do nursing students experience the clinical learning environment and respond to their experiences? A qualitative study. BMJ Open. 2019;9(7):e028052. 31350243 10.1136/bmjopen-2018-028052 PMC6661598

[51] Hosseini SH, Tayebi Z, Poormoosa Poostin Saraee Y. Nursing students’ perception of nurses’ professional misconduct: a descriptive qualitative study. JMED. 2024;17(53):34–44.

[52] Curtis DA, Braziel JM, Redfearn RA, Hall J. Lying to patients: Ethics of deception in nursing. Clin Ethics. 2020;16(4):341–6. 10.1177/1477750920977103

[53] Pritchard J. Using therapeutic lies – an ethical challenge for nurses when caring for people with dementia. Nurs Older People; 2024. 10.7748/nop.2024.e1468 38444165

[54] Goethals S, Gastmans C, de Casterlé BD. Nurses’ ethical reasoning and behaviour: a literature review. Int J Nurs Stud. 2010;47(5):635–50. 20096413 10.1016/j.ijnurstu.2009.12.010

[55] Tanaka M. Thoughts and feelings that determine how Japanese nursing students deal with ethical issues: a qualitative study. Int J Ethics Educ. 2021;6(2):323–37. 10.1007/s40889-021-00127-1

[56] Arries EJ. Professional values and ethical ideology: perceptions of nursing students. Nurs Ethics. 2020;27(3):726–40. 31829088 10.1177/0969733019889396

[57] Engel J, Salfi J, Micsinszki S, Bodnar A. Informed strangers: Witnessing and responding to unethical care as Student nurses. Glob Qual Nurs Res. 2017;4:2333393617730208. 28932765 10.1177/2333393617730208 PMC5600298

