# Supplementary material for: Nurturing ethical insight: exploring nursing students’ journey to ethical competence
Source: BMC Nurs. 2024 Aug 15;23:568. doi: 10.1186/s12912-024-02243-x (PMC11328478; doi:10.1186/s12912-024-02243-x)
Supplement: Supplementary file 1 — Supplementary Material 1 [file 12912_2024_2243_MOESM1_ESM.docx]

**Supplementary file 1.** Interview guide

| 1. **Learning to identify ethical problems in clinical studies** Entrance- and follow-up questions: 2. Please provide examples of ethical problems that you experienced on your ward. Describe what happened, who was involved, how the involved parties were affected and their perspectives, how you were affected and your viewpoints, and what you found challenging. 3. What made you sense that the situations were ethically problematic? Did you perceive ethical tension yourself, were you influenced by what the residents said or did, did your peers, nurse preceptor, or others guide your perceptions? 4. What kind of guidance did you feel you needed to identify ethical problems? 5. How well did your clinical studies prepare you to identify ethical problems? 6. In your opinion, which learning experiences contributed the most to your understanding of ethically complex situations? 7. **Acquiring ethical knowledge and skills in clinical studies**   Entrance- and follow-up questions:   1. Please provide examples of ethical knowledge you learned and applied in your clinical studies. You may include ethical principles and their application in situations, nurses’ ethical guidelines, relevant legislation, knowledge of appropriate conduct, communication methods, and any other relevant aspects. 2. How did you learn to apply ethical knowledge? Was it through reading, being instructed, hands-on practice, experiencing real situations, noticing the importance of certain actions, sensing something was wrong or concerning, or feeling a duty on behalf of residents? 3. In your opinion, what is important to learn in practice to ensure high ethical standards in nursing? 4. Do you think your clinical studies were well-suited for acquiring ethical knowledge and skills? Why or why not? 5. **Learning to assess and reflect on the best or most appropriate solutions to ethical problems in clinical studies**   Entrance- and follow-up questions:   1. Please provide examples of solutions to ethical problems addressed on your ward. You may describe the reflections, assessments, and choices made, who was involved, how and when you were involved, whether the assessments and interventions were meaningful to you, and if you found the resolutions to be in the residents’ best interest, and why or why not. 2. How did the residents respond to the resolutions implemented? 3. In a similar situation, would you now solve the ethical problem in the same manner? What would you do differently, and why would you choose a different approach? 4. Did you assess and reflect on solutions to ethical problems on your own, or did you collaborate with others? If so, who did you work with? 5. In your opinion, did you receive sufficient supervision and support in assessing the best or most appropriate solutions to ethical problems? 6. How would you describe the contribution of your clinical studies to your learning in assessing and implementing solutions to ethical problems?   **Eventually:**  If not previous discussed in the focus group, please address:   1. How do you think your clinical studies contributed to the development of your ethical competence? Please provide examples. 2. How is the development of ethical competence in university courses different from the development in clinical studies? Specifically, how did your clinical studies contribute to your ethical competence? |
| --- |
